# Catheter ablation and thoracoscopic ablation in long persistent atrial fibrillation with large left atrium

**DOI:** 10.3389/fcvm.2022.881831

**Published:** 2022-09-23

**Authors:** Chan Soon Park, Eue-Keun Choi, So-Ryoung Lee, Hyo-Jeong Ahn, Soonil Kwon, Sunhwa Kim, Suk Ho Sohn, Jae Woong Choi, Ho Young Hwang, Seil Oh

**Affiliations:** ^1^Department of Internal Medicine, Seoul National University Hospital, Seoul, South Korea; ^2^Department of Internal Medicine, Seoul National University College of Medicine, Seoul, South Korea; ^3^Department of Thoracic and Cardiovascular Surgery, Seoul National University Hospital, Seoul, South Korea; ^4^Department of Thoracic and Cardiovascular Surgery, Seoul National University College of Medicine, Seoul, South Korea

**Keywords:** atrial fibrillation, enlarged left atrium, radiofrequency catheter ablation, cryoablation, thoracoscopic maze, recurrence

## Abstract

**Background:**

Pulmonary vein antrum isolation (PVAI) is the cornerstone of atrial fibrillation (AF) ablation, but the clinical outcomes of PVAI are unsatisfactory in patients with persistent AF and a large left atrium (LA).

**Objectives:**

We investigated the clinical outcomes following radiofrequency ablation (RFCA), cryoballoon ablation (CBA), and thoracoscopic maze in patients with persistent AF and a large LA.

**Methods:**

We included patients with consecutive persistent AF who had a large LA (LA diameter >50 mm) and underwent RFCA, CBA, or thoracoscopic maze surgery. In the RFCA group, additional linear ablation was performed at the physician’s discretion. The endpoint was 12 months without recurrence of an atrial arrhythmia, including AF, atrial flutter, and atrial tachycardia, following a 90-day blanking period.

**Results:**

We recruited 89 persistent AF patients with a large LA who underwent RFCA (*n* = 32), CBA (*n* = 38), or the thoracoscopic maze procedure (*n* = 19). During the 12-month follow-up, 48 (53.9%) cases of AF recurrence were observed. There was no prognostic difference between groups (50.0% in RFCA vs. 52.6% in CBA vs. 63.2% in thoracoscopic maze, all *P* > 0.05). Early recurrence during the blanking period was a significant predictor of late recurrence for RFCA and CBA, but not for the thoracoscopic maze.

**Conclusion:**

In persistent AF patients with a large LA, we did not find a prognostic difference RFCA, CBA, or a thoracoscopic maze procedure in recurrence of atrial arrhythmia. Early recurrence predicted late recurrence in catheter ablation, but not in thoracoscopic maze.

## Introduction

Atrial fibrillation (AF) is the most common clinical arrhythmia and is associated with substantial morbidity and mortality rates worldwide (1, 2). Due to the associated disease burden, various attempts have been made to improve the prognosis for patients with AF. During the past decade, the clinical outcomes following interventional treatment of AF have improved. Catheter ablation is now considered a well-established treatment for AF management, and pulmonary vein antrum isolation (PVAI) with catheter ablation has become the cornerstone approach to AF ablation (3–5). Radiofrequency catheter ablation (RFCA) can produce superior sinus rhythm maintenance rates over those of anti-arrhythmic drug medications (6, 7). Based on the beneficial effects of catheter ablation, the current guidelines recommend such when rhythm control is selected for long-term management of patients with AF (4).

In recent randomized trials, cryoballoon ablation (CBA) showed similar outcomes to those of RFCA in managing patients with AF; arrhythmia-free survival and complication rates were comparable between the two energy sources (8–10). In addition, the thoracoscopic maze procedure has been shown to have a lower recurrence rate of AF than that of RFCA (11), but with higher complication rates and longer hospitalizations (12, 13). However, previous studies that investigated the favorable outcomes of these ablation strategies mainly included patients with paroxysmal AF (8, 9, 14, 15), those with persistent AF without long-standing AF (>1 year) (7, 16), or AF patients with a limited size of the left atrium (LA) (7–9). Indeed, the clinical outcomes of PVAI in chronic AF patients with a large LA are unsatisfactory and less available (17, 18). Therefore, it remains unclear which interventional strategy would be preferred for managing persistent AF patients with a large LA (4, 5).

Therefore, we sought to investigate the clinical outcomes of interventional approaches to rhythm control, including RFCA, CBA, and the thoracoscopic maze procedure, in patients with long-persistent AF with a large LA.

## Materials and methods

### Study population and data collection

We screened patients with persistent AF who had a large LA (left atrial diameter >50 mm) and underwent RFCA, CBA, or thoracoscopic maze surgery. We excluded those with a previous history of AF ablation. We enrolled patients with RFCA and CBA between April 2019 and May 2020 as CBA was first introduced and has been performed to Seoul National University Hospital since 2019. Patients with previous atrial flutter were treated with RFCA instead of CBA for cavotricuspid isthmus (CTI) ablation. As only five patients underwent thoracoscopic maze surgery during this period, patients undergoing the thoracoscopic maze procedure were recruited between August 2016 and May 2020. Baseline characteristics, including demographic data, underlying diseases, echocardiographic data, and medication history, were retrieved from electronic medical records. Echocardiographic assessments were performed within 1 month preceding the interventional treatment. This study was conducted in accordance with the Declaration of Helsinki and was approved by the institutional review board of Seoul National University Hospital (IRB No. H-2108-190-1248).

### Ablation procedures

RFCA was performed using available irrigation catheters from the Navistar Thermocool SF/Thermocool Smart Touch (Biosense Webster). Electroanatomical maps of the atria and pulmonary veins were created via three-dimensional mapping with the CARTO−3 (Biosense Webster). All patients initially underwent circumferential PVAI. An additional bidirectional block of the CTI was performed if the patient had a previous history of atrial flutter. Additionally, a roof line, posteroinferior line, mitral isthmus line, and/or anterior line were also performed at the discretion of the electrophysiologist. The targeted ablation index was previously reported elsewhere (19, 20). The point-by-point RFCA applications were delivered using the power-controlled mode with 30–40 W (irrigation flow up to 15 mL/min) at the anterior/roof segments and 25–30 W (irrigation flow up to 8 mL/min) at the posterior/inferior/carina segments. Briefly, RF energy was delivered until an AI of ≥450 was attained at the anterior/roof segments, and an AI of ≥350 was attained at the posterior/inferior/carina segments. The targeted catheter force was between 5 and 20 g. Each annotation point was presented according to the ablation index as a lesion tag size of 2 mm (radius 2 mm ball), and the maximal interlesion distance between neighboring lesions was ≤4 mm. If the catheter dislocated before reaching the target AI, a new RF ablation was applied to reach the ablation index target.

CBA was performed for PVAI using the Arctic Front Advance Catheter (Medtronic) and Achieve Mapping catheter (Medtronic). During CBA, superior vena cava (SVC) pacing was performed to detect phrenic nerve injury during right-sided PVAI. Optimal PV occlusion was confirmed by contrast media injection through the catheter. Then, PVAI was performed by freezing each PV antrum upto 240 s. If PVAI was not achieved during 90 s of CBA, we terminated the freeze and repositioned the catheter for an optimal PV occlusion. After the CBA, we confirmed the entrance and exit block of the PV with a Achieve Mapping catheter.

Thoracoscopic maze surgery was performed using a bilateral thoracoscopic approach as previously described (21, 22). Briefly, the procedure was performed under general anesthesia. In addition to PVAI, LA lines, including both superior and inferior lines, were created, and the ligament of Marshall was divided. The ganglionated plexus was ablated, and the left atrial appendage was removed or obliterated—a selectively performed ablation of the trigone (aortic root line) and line from the SVC to the IVC. In the RFCA group and CBA group, left atrial appendage occlusion was not performed.

### Clinical outcomes and variables

The endpoint of this study was late recurrence of atrial arrhythmia during the 12-month follow-up. Late recurrence was defined as a ≥30-s recurrence of atrial arrhythmia, including AF, atrial flutter, and atrial tachycardia following a 90-day blanking period. Atrial arrhythmia during the blanking period was defined as early recurrence and was not considered an endpoint. Patients were managed according to the standard of care based on clinical guidelines (4, 5). If AF persisted after ablation, internal or external electrical cardioversion was performed. When atrial arrhythmia occurred during the blanking period, cardioversion was performed. To detect recurrence of atrial arrhythmia, follow-up visits were arranged after the blanking period from 3, 6, 9, and 12 months. A 12-lead electrocardiogram was performed at each follow-up visit, and a 24-h Holter monitoring was performed at 3- and 12-month visits. Patients were prescribed anti-arrhythmic drug medications after the interventional rhythm control according to the physician’s discretion. For safety outcomes, we collected data on procedure-related complications including steam pop, major bleeding requiring intervention, atrial-esophageal fistula, phrenic nerve palsy, cardiac tamponade, thromboembolic events, complications that require unplanned intervention, and deaths up to 12 months. Thromboembolic events include stroke, TIA and other thromboembolic events. Vascular events are defined as prior myocardial infarction, peripheral artery disease and aortic plaque (23).

### Statistical analysis

Categorical variables were presented as numbers and frequencies, and continuous variables were expressed as mean ± standard deviation or median with interquartile ranges. For comparison between groups, the chi-square test (or Fisher’s exact test when any expected cell count was <5 for a 2 × 2 table) was used for categorical variables, and the unpaired Student’s *t*-test for continuous variables. The chronological trend of outcomes was expressed in Kaplan–Meier estimates and compared to that of the interventional treatment. The log-rank test was performed to compare the differences in clinical outcomes between the two groups. The Cox proportional hazard regression model was used to determine prognostic significance of each variable for RFCA, CBA, and thoracoscopic maze surgery. Two-sided *P*-values <0.05 were considered statistically significant. Statistical tests were performed using IBM SPSS statistics version 25 (SPSS Inc., Chicago, IL, USA).

## Results

### Baseline characteristics of the study population

We included 89 persistent AF patients with a large LA (left atrial diameter >50 mm) in this study; 32 (36.0%) patients underwent RFCA, 38 (42.7%) underwent cryoablation, and 19 (21.3%) underwent the thoracoscopic maze procedure during the recruitment period. The mean age was 63.5 ± 6.8 years, 79.8% were male, 16.9% had heart failure, 69.7% had hypertension, and 21.3% had diabetes mellitus. Among the study population, 70.8% had a history of direct current cardioversion for rhythm control. Before ablation, patients were diagnosed with AF for 4.2 ± 3.7 years (median 2.7 years with interquartile range 1.3–5.9 years).

The baseline characteristics of the study population are presented in Table 1. Patients who underwent RFCA or CBA had a more frequent history of diabetes mellitus than those who underwent the thoracoscopic maze surgery (28.1 vs. 26.3 vs. 0.0%, *P* = 0.037). Consequently, patients who underwent the thoracoscopic maze procedure had a more frequent history of thromboembolic events (3.1 vs. 0.0 vs. 15.8%, *P* = 0.023), and larger LA size (54.5 ± 3.7 mm vs. 53.8 ± 2.4 mm vs. 58.1 ± 4.8 mm for RFCA, CBA, and thoracoscopic maze, *P* < 0.001). There was no statistically significant difference in the prevalence of heart failure and hypertension across the treatment groups. At 3-month visits and 12-month visits, the 24-hour Holter monitoring was performed equivalently across the treatment groups (*P* = 0.822 and *P* = 0.670, respectively).

**TABLE 1:** Baseline characteristics of PeAF patients with large LA.

	RFCA (*n* = 32)	CBA (*n* = 38)	Thoracoscopic maze (*n* = 19)	*P*-value
**Demographic data**				
Age (years)	64.2 ± 5.6	64.8 ± 6.0	59.8 ± 8.9	0.023
Men (%)	21 (65.6)	34 (89.5)	16 (84.2)	0.040
BMI (kg/m^2^)	25.9 ± 3.4	26.1 ± 2.5	26.2 ± 3.1	0.917
**Past medical history (%)**				
Heart failure	4 (12.5)	8 (21.1)	3 (15.8)	0.629
Hypertension	22 (68.8)	28 (73.7)	12 (63.2)	0.710
Diabetes mellitus	9 (28.1)	10 (26.3)	0 (0)	0.037
Thromboembolic events	1 (3.1)	0 (0.0)	3 (15.8)	0.023
Vascular events	0 (0.0)	0 (0.0)	0 (0)	N/A
CHA*2*DS*2*-VASc	2.0 ± 1.4	1.9 ± 0.9	2.5 ± 1.2	0.171
**Atrial fibrillation history**				
Years diagnosed with AF	4.0 ± 3.2	4.4 ± 4.2	4.1 ± 3.6	0.928
Previous DCC (%)	24 (75.0)	22 (57.9)	17 (89.5)	0.038
**Echocardiography**				
LAD (mm)	54.5 ± 3.7	53.8 ± 2.4	58.1 ± 4.8	<0.001
LAvolume index (ml/m^2^)	66.9 ± 20.7	66.2 ± 15.9	80.6 ± 19.3	0.032
LVEF (%)	55.1 ± 8.7	58.0 ± 6.7	60.3 ± 5.5	0.043
**Medications at discharge (%)**				
Class Ic drug	13 (40.6)	11 (28.9)	2 (10.5)	0.073
Class III drug	10 (31.3)	21 (55.3)	16 (84.2)	0.001
Beta-blocker	11 (34.4)	10 (26.3)	7 (36.8)	0.655
Calcium channel blocker	3 (9.4)	2 (5.3)	7 (36.8)	0.003
**Holter monitoring during follow up (%)**				
3-month visits	17 (53.1)	23 (60.5)	11 (57.9)	0.822
12-month visits	14 (43.8)	14 (36.8)	6 (31.6)	0.670
At least once	22 (68.8)	26 (68.4)	11 (61.1)	0.835
**Medications at 3 month follow-up (%)**				
Class Ic drug	12 (31.6)	6 (18.8)	4 (21.1)	0.425
Class III drug	17 (44.7)	8 (25.0)	16 (84.2)	<0.001
Beta-blocker	13 (34.2)	14 (43.8)	5 (26.3)	0.436
Calcium channel blocker	3 (7.9)	4 (12.5)	8 (42.1)	0.004
**Recurrence (%)**				
Early recurrence	16 (50.0)	21(55.3)	17 (89.5)	0.014

AF, atrial fibrillation; BMI, body mass index; CBA, cryoballon ablation; DCC, direct current cardioversion; LA, left atrium; LVEF, left ventricular ejection fraction; RFCA, radiofrequency ablation.

### Clinical outcomes according to treatment strategy

During the blanking period, patients who underwent the thoracoscopic maze procedure had higher early recurrence rates during the blanking period (50.0 vs. 55.3 vs. 89.5% for RFCA, CBA, and thoracoscopic maze, *P* = 0.014). During the 12-month follow-up, 48 (53.9%) cases of late recurrence were observed. As shown in Supplementary Table 1, no statistical difference was observed between those with and without late recurrence in terms of age, sex, and previous history of heart failure, hypertension, diabetes mellitus, thromboembolic events, and CHA*2*DS*2*-VASc score. In contrast, patients with late recurrence showed higher left ventricular ejection fraction levels and early recurrence rate during the blanking period than those without late recurrence. Supplementary Table 2 shows the use of anti-arrhythmic drugs in 48 patients with late recurrence at the time of recurrence. Among patients with late recurrence, nine patients underwent redo procedures (three patients in the CBA group, three patients in RFCA group, and three patients in the thoracoscopic maze surgery group) during the 12-month follow-up. These patients were all treated with redo RFCA. Among these nine patients, PV reconnection was observed in eight subjects. Additional ablation to PVAI was performed in seven patients (LA roof line in six patients, posteroinferior line in two patients, CTI in two patients, and within the posterior wall in three patients), and six patients experienced a recurrence of AF within 12 months after redo RFCA.

**FIGURE 1:**
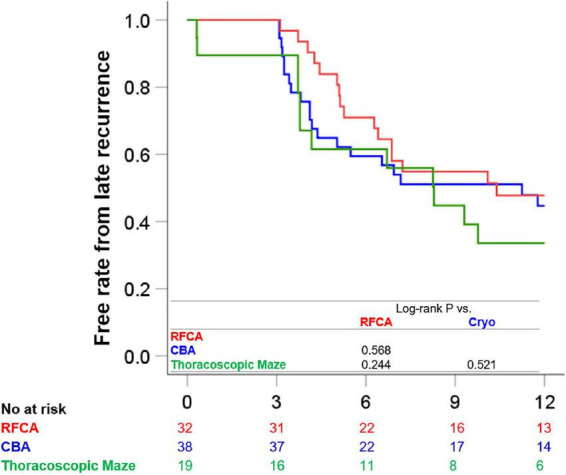
Clinical outcomes according to each treatment strategy. The Kaplan-Meier survival curves for freedom from 12-month late recurrence in patients treated with RFCA, those with CBA, and those treated with thoracoscopic maze surgery are presented. CBA, cryoballoon ablation; RFCA, radiofrequency catheter ablation.

Kaplan-Meier recurrence free survival curves for each ablation strategy are presented in Figure 1. There were no prognostic differences between RFCA and CBA (late recurrence rates 50.0 vs. 52.6%, Log-rank *P* = 0.568), between RFCA and thoracoscopic maze (late recurrence rates 50.0 vs. 63.2%, log-rank *P* = 0.244), and between CBA and thoracoscopic maze (late recurrence rates 52.6 vs. 63.2%, log-rank *P* = 0.521). After adjusting for age and LA diameter, there was still no prognostic difference across ablation strategies (between RFCA and CBA, *P* = 0.566, between RFCA and thoracoscopic maze, *P* = 0.402, and between CBA and thoracoscopic maze, *P* = 0.933).

We further stratified patients with RFCA into PVAI only (*n* = 17) vs. PVAI with additional ablation (*n* = 15). CTI ablation was the most frequently performed extra-ablation (12/15). Detailed information on additional ablation is presented in Supplementary Table 3. In brief, ablation of the CTI was most frequently performed (12/15, 80%), and LA roof line (5/15, 33.3%), LA inferior line (2/15, 13.3%), and other ablations were also performed according to the electrophysiologist’s decision. Patients with RFCA PVAI with additional ablation had similar baseline characteristics, except for male preponderance, decreased history of hypertension, and lower CHA2DS2-VASc scores (Supplementary Table 4). Supplementary Figure 1A shows chronological trends of AF recurrence when patients were stratified with those RFCA PVAI with additional ablation, patients with PVAI only (RFCA or CBA), and patients with thoracoscopic maze surgery. Baseline characteristics and clinical outcomes when patients were stratified into RFCA PVAI, RFCA PVAI with additional ablation, CBA, and thoracoscopic maze surgery are presented in the Supplementary Figure 1B. Among included, one patient treated with thoracoscopic maze surgery was suffered from thromboembolic stroke 1-month after operation and died.

When patients were stratified according to the use of any anti-arrhythmic drug medications or according to the use of class Ic drugs and class III drugs, no prognostic difference was observed (Log-rank *P* = 0.301 and Log-rank *P* = 0.806, respectively).

### Predictors for atrial fibrillation recurrence according to ablation strategies

**FIGURE 2:**
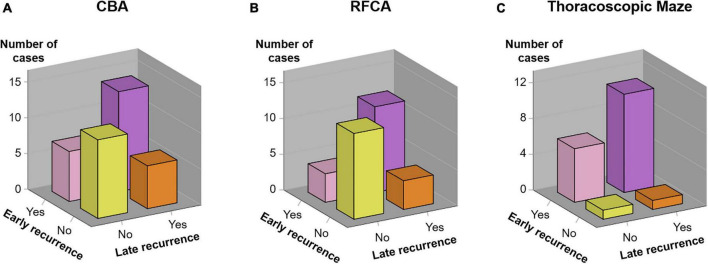
Association between early and late recurrence. The incidence of early recurrence and late recurrence in each treatment group [**(A)** CBA group, **(B)** RFCA group, and **(C)** Thoracoscopic Maze group] is demonstrated. CBA, cryoballoon ablation; RFCA, radiofrequency catheter ablation.

We investigated parameters that could predict AF according to ablation strategies: age, sex, CHA*2*DS*2*-VASc score, years diagnosed with AF, LA diameter, and early recurrence (Table 2). For the RFCA group, early recurrence showed significant prognostic value (hazard ratio [HR] 4.698, 95% confidence interval [CI] 1.498–14.738, *P* = 0.008), while other factors did not. Similarly, early recurrence was predictive for late recurrence in the CBA group (HR 3.410, 95% CI 1.291–9.007, *P* = 0.013). However, neither early recurrence nor other factors showed significant predictive power in the thoracoscopic maze group. The proportion of patients diagnosed with early and late recurrence in each group is shown in Figure 2.

**TABLE 2:** Cox regression analyses to evaluate prognostic implication of each variable (univariate analysis for RFCA, CBA, and thoracoscopic maze).

Parameters	Hazard ratio	95% confidence interval	*P*-value
**RFCA**			
Age (years)	1.036	0.953–1.126	0.403
Male	0.574	0.213–1.543	0.271
CHA*2*DS*2*-VASc score (points)	1.150	0.838–1.578	0.387
Years diagnosed with AF (years)	1.005	0.850–1.189	0.950
LA diameter (mm)	0.936	0.810–1.081	0.367
Early recurrence	4.698	1.498–14.738	0.008
**CBA**			
Age (years)	1.025	0.949–1.107	0.535
Male	0.357	0.118–1.079	0.068
CHA*2*DS*2*-VASc score (points)	1.040	0.637–1.698	0.874
Years diagnosed with AF (years)	1.046	0.952–1.148	0.349
LA diameter (mm)	0.967	0.806–1.161	0.722
Early recurrence	3.410	1.291–9.007	0.013
**Thoracoscopic maze**			
Age (years)	1.009	0.947–1.075	0.775
Male	1.692	0.218–13.148	0.615
CHA*2*DS*2*-VASc score (points)	1.051	0.689–1.604	0.816
Years diagnosed with AF (years)	0.976	0.805–1.184	0.808
LA diameter (mm)	1.079	0.947–1.229	0.252
Early recurrence	1.560	0.201–12.120	0.671

AF, atrial fibrillation; CBA, cryoballoon ablation; LA, left atrium; RFCA, radiofrequency ablation.

## Discussion

The main findings of our study were as follows: (1) among patients with persistent AF and a large LA, more than half experienced late recurrence after ablation during the 12-month follow up; (2) rhythm control intervention strategies, including RFCA, CBA, and the thoracoscopic maze procedure, did not show a significant difference in AF recurrence; (3) early recurrence was a significant predictor of late recurrence in patients who underwent RFCA or CBA, but not in those treated with the maze procedure.

For patients with persistent AF, extra-ablation in addition to PVAI has been shown to achieve better clinical benefits from RFCA (24, 25). In patients who were previously diagnosed with CTI-dependent atrial flutter, CTI ablation can prevent atrial flutter (24, 26). To decrease the risk of AF recurrence, linear ablation of the left atrium, ablation of complex fractionated electrograms, isolation of the SVC or left atrial appendage, ablation to non-pulmonary foci, etc., can be considered (25, 27–29). In the past decade, adding an extra-ablation to PVAI during the first ablation procedure has proven controversial (30, 31). Notably, the Substrate and Trigger Ablation for Reduction of Atrial Fibrillation Trial Part II (STAR AF II) study showed that neither linear ablation nor ablation of complex fractionated electrograms reduced recurrence in patients with persistent AF (32). However, previous studies have mainly focused on persistent AF with a relatively small LA. The STAR AF II study and the Ablation at St. Georg Hospital for Long-Standing Persistent Atrial Fibrillation study (Alster-Lost-AF study) excluded patients with persistent AF who had a long history of AF or larger LA (31, 32). Another study also reported that LA posterior wall isolation, in addition to PVAI, did not improve outcomes compared to PVAI alone (30). While this study enrolled both paroxysmal and persistent AF patients, paroxysmal AF patients accounted for the majority (60%) of the study population. These studies also provided additional ablation to the standardized protocol after randomization. In contrast, recent studies have shown that individually tailored substrate modification, rather than additional standardized ablation, could provide a better prognosis (33, 34), which might explain the limited therapeutic efficacy of additional ablation in previous reports. Due to limited number of included subjects, whether tailored substrate modification could provide more benefits in patients with persistent AF and a large LA remains undetermined in this study.

CBA is another well-established interventional treatment for AF (4). Various studies based on real-world registries have suggested that CBA might be a safe, effective, and efficient treatment strategy for persistent AF (17, 35). However the trials showing that CBA could be applied in patients with persistent AF with similar results to those of RFCA were small, and additional randomized studies to verify the value of CBA in persistent AF are necessary (36). The maze procedure, a surgical approach to treating AF, has produced notable advances in the management of AF patients. Thoracoscopic maze surgery has generated remarkable therapeutic results (11), and reportedly can lower AF recurrence rates but has higher complication rates than those of catheter ablation (37, 38). Indeed, however, there is a paucity of data regarding treatment of patients with persistent AF, a long history and large LA size, who are prone to frequent AF recurrence. To answer this unanswered question, we explored the clinical outcomes of RFCA, CBA, and thoracoscopic maze surgery in patients with high recurrence risks.

AF recurrence is frequently observed during the first 3 months after ablation (39). Inflammation caused by catheter ablation has been suggested as a major cause of early recurrence. There is a consensus that AF recurrence during the blanking period is not a true AF recurrence (4). Despite the concept of a blanking period, however, early recurrence has proven to be predictive of late recurrence of AF (39, 40). In this study, the risks of late recurrence were equivalently observed across the RFCA, CBA, and thoracoscopic maze treatment groups, whereas the risk of early recurrence was significantly higher in the thoracoscopic maze group. Furthermore, early recurrence was a significant predictor of late recurrence in the RFCA and CBA groups, but not in the thoracoscopic maze groups. This suggests that the transient inflammatory status of LA after RFCA or CBA might be different from that of LA after thoracoscopic maze. Authors acknowledge that careful consideration should be demanded as thoracoscopic maze group was prescribed with class III anti-arrhythmic agents more frequently.

This study provided important clinical information. In spite of robust evidences for benefits of interventional rhythm control strategies, there is a paucity of data regarding outcomes in patients with persistent AF and a large LA. In this study, we included patients with persistent AF and a larger LA, who have rarely been included in previous reports. We also analyzed and compared the therapeutic efficacy of RFCA, CBA, and thoracoscopic maze surgery during the concurrent period. We found that persistent AF patients with a larger LA received similar benefits from RFCA, CBA, and thoracoscopic maze. This finding does not negate the need for clinicians to focus on ablation strategies. In contrast, we suggest that treatment strategies should be carefully adapted according to each patient’s clinical characteristics.

This study has several limitations. This was a retrospective cohort study with a small number of patients, hence, larger, prospective studies are warranted to validate our findings. Accordingly, limited statistical power was found in this manuscript (power = 0.50, alpha = 0.05, and two-sided design). Indeed, we only analyzed 19 patients who underwent thoracoscopic maze surgery with a longer inclusion period. The authors acknowledge that there is a possibility of unmeasured confounding variables and biases and that the statistical power was limited. For example, proportion of patients taking class III anti-arrhythmic agents was different across groups, and careful consideration should be demanded. However, this study suggests the necessity of further studies regarding long-persistent AF patients with a large LA; a larger prospective cohort or a randomized trial is required to verify our results with substantial statistical power. Second, the subjects included in this study were limited to East Asian patients; extrapolation to other ethnicities needs further investigation.

## Conclusion

RFCA, CBA, or the thoracoscopic maze procedure did not find a prognostic difference among patients with persistent AF and a large LA. Early recurrence predicted late recurrence in patients treated with catheter ablation, but not in those treated with thoracoscopic maze.

## Data availability statement

For reasonable request, data would be available through approval of the corresponding author.

## Ethics statement

The studies involving human participants were reviewed and approved by institutional review board of Seoul National University Hospital. Written informed consent for participation was not required for this study in accordance with the national legislation and the institutional requirements.

## Author contributions

CSP and E-KC conceptualized the study, were responsible for review and editing. CSP drafted the manuscript. CSP, E-KC, S-RL, H-JA, SKw, SKi, SHS, JWC, HYH, and SO contributed in data acquisition. CSP, E-KC, S-RL, H-JA, SKw, and SKi interpreted the data. CSP, E-KC, and SO supervised the project. All authors contributed to the article and approved the submitted version.
