# Supplementary material for: Catheter ablation and thoracoscopic ablation in long persistent atrial fibrillation with large left atrium
Source: Front Cardiovasc Med. 2022 Sep 23;9:881831. doi: 10.3389/fcvm.2022.881831 (PMC9537550; doi:10.3389/fcvm.2022.881831)
Supplement: Supplementary file 1 [file Data_Sheet_1.docx]

**
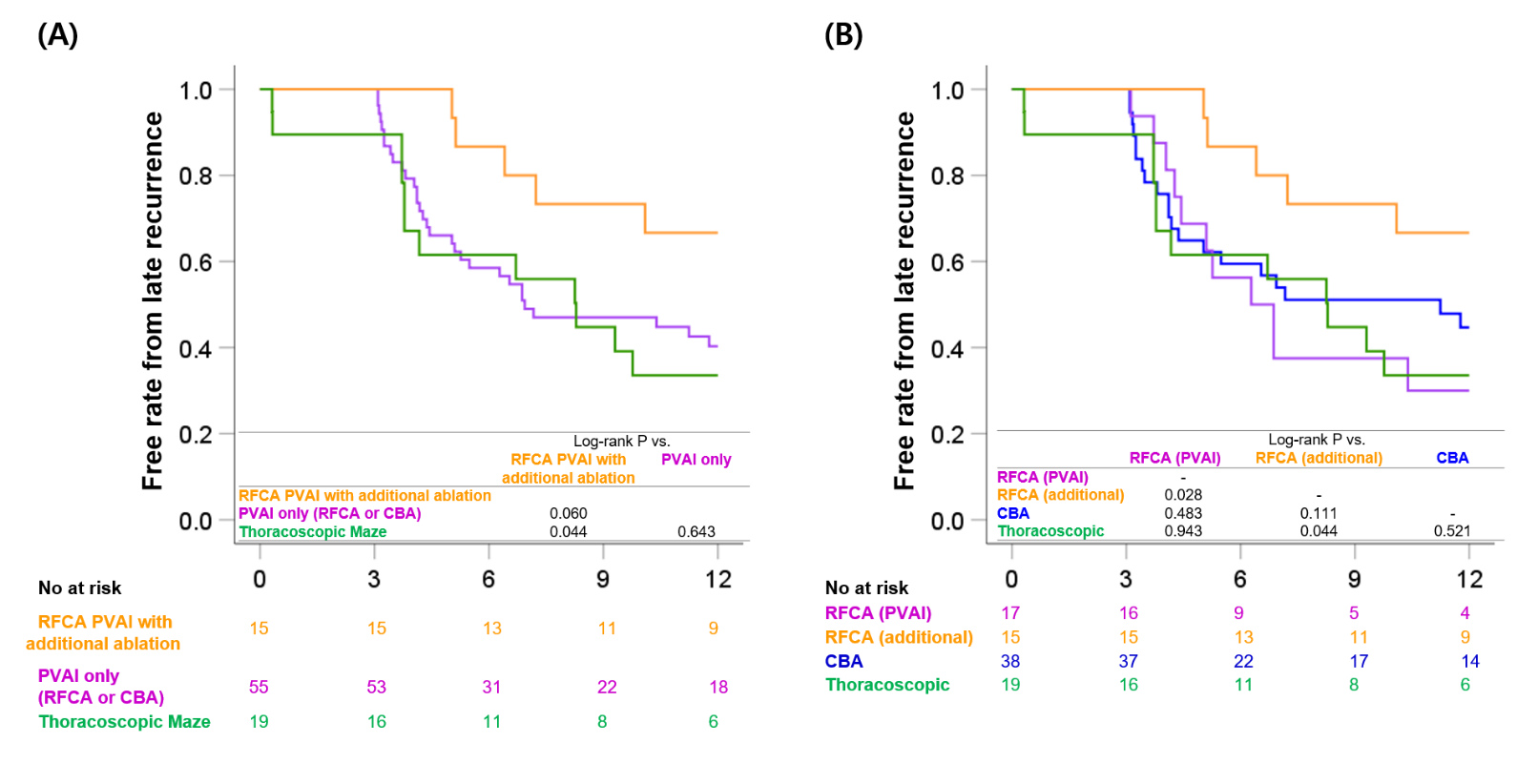
**

**Supplemental Figure 1.** **Clinical outcomes according to RFCA PVAI, RFCA PVAI with additional ablation, CBA, and thoracoscopic maze surgery**

(A) Clinical outcomes are illustrated when patients were stratified into RFCA PVAI with additional ablation group, PVAI only group (RFCA or CBA), and the thoracoscopic maze group. (B) The Kaplan-Meier survival curves for freedom from 12-month late recurrence in patients treated with RFCA PVAI, those with RFCA PVAI and additional ablation, those with CBA, and those treated with thoracoscopic maze surgery are presented.

CBA, cryoballoon ablation; PVAI, pulmonary vein antrum isolation; RFCA, radiofrequency catheter ablation

**Supplemental Table 1**. Baseline characteristics according to atrial arrhythmia recurrence

|  | **Without late recurrence**  **(n=41)** | **With late recurrence**  **(n=48)** | **P-value** |
| --- | --- | --- | --- |
| **Demographic data** |  |  |  |
| Age (years) | 63.6±7.3 | 63.5±6.4 | 0.944 |
| Men (%) | 35 (85.4) | 36 (75.0) | 0.225 |
| BMI (kg/m^2^) | 26.5±3.0 | 25.7±2.9 | 0.259 |
| **Past Medical History (%)** |  |  |  |
| Heart Failure | 8 (19.5) | 7 (14.6) | 0.536 |
| Hypertension | 28 (68.3) | 34 (70.8) | 0.795 |
| Diabetes mellitus | 9 (22.0) | 10 (20.8) | 0.898 |
| Thromboembolic events | 2 (4.9) | 2 (4.2) | 0.872 |
| Vascular events | 0 (0) | 0 (0) | N/A |
| CHA₂DS₂-VASc | 1.9±1.2 | 2.2±1.1 | 0.376 |
| **Atrial fibrillation history** |  |  |  |
| Years diagnosed with AF | 4.2±3.7 | 4.2±3.7 | 0.974 |
| Previous DCC (%) | 31 (75.6) | 32 (66.7) | 0.355 |
| **Echocardiography** |  |  |  |
| LAD (mm) | 54.9±3.9 | 55.1±3.7 | 0.765 |
| LA volume index (ml/m^2^) | 65.0±17.7 | 72.7±19.6 | 0.069 |
| LVEF (%) | 55.3±7.4 | 59.2±7.2 | 0.014 |
| **Medications at discharge (%)** |  |  |  |
| Class Ic drug | 9 (22.0) | 17 (35.4) | 0.164 |
| Class III drug | 24 (58.5) | 23 (47.9) | 0.317 |
| Beta-blocker | 12 (29.3) | 16 (33.3) | 0.681 |
| Calcium channel blocker | 5 (12.2) | 7 (14.6) | 0.742 |
| **Recurrence (%)** |  |  |  |
| Early recurrence | 17 (41.5) | 37 (77.1) | 0.001 |

AF, atrial fibrillation; BMI, body mass index; DCC, direct current cardioversion; LA, left atrium; LR, late recurrence; LVEF, left ventricular ejection fraction; RFCA, radiofrequency ablation

**Supplemental Table 2. Use of Anti-arrhythmic drugs at late recurrence**

| **Medications at late recurrence** |  |
| --- | --- |
| Class Ic drug | 16 (33.3%) |
| Class III drug | 18 (37.5%) |
| Beta-blocker | 19 (39.6%) |
| Calcium channel blocker | 10 (20.8%) |

**Supplemental table 3. Additional ablation in addition to RFCA**

| **Ablation** | **N (% from overall RFCA group)** |
| --- | --- |
| All | 15 (46.9) |
| LA roof | 5 (15.6) |
| LA inferior | 2 (6.3) |
| CTI | 12 (37.5) |
| SVC | 0 (0.0) |
| etc | 2 (0.3) |

AF, atrial fibrillation; CTI, cavotricuspid isthmus; LA, left atrium; RFCA, radiofrequency ablation

**Supplemental Table 4**. **Baseline characteristics of subjects according to extra-ablation in addition to PVAI**

|  | **RFCA PVAI with additional ablation**  **(n=15)** | **PVAI only**  **(RFCA and CBA)**  **(n=53)** | **Thoracoscopic maze (n=19)** | **P-value** |
| --- | --- | --- | --- | --- |
| **Demographic data** |  |  |  |  |
| Age (years) | 62.3±5.2 | 65.1±5.8 | 59.8±8.9 | 0.009 |
| Men (%) | 13 (86.7) | 42 (76.4) | 16 (84.2) | 0.586 |
| BMI (kg/m^2^) | 26.5±4.2 | 25.9±2.5 | 26.2±3.1 | 0.778 |
| **Past Medical History (%)** |  |  |  |  |
| Heart Failure | 2 (13.3) | 10 (18.2) | 3 (15.8) | 0.897 |
| Hypertension | 7 (46.7) | 43 (78.2) | 12 (63.2) | 0.049 |
| Diabetes mellitus | 3 (20.0) | 16 (29.1) | 0 (0) | 0.028 |
| Thromboembolic events | 1 (6.7) | 0 (0.0) | 3 (15.8) | 0.015 |
| Vascular events | 0 (0.0) | 0 (0.0) | 0 (0) | N/A |
| CHA₂DS₂-VASc | 1.5±1.5 | 2.1±1.0 | 2.5±1.2 | 0.040 |
| **Atrial fibrillation history** |  |  |  |  |
| Years diagnosed with AF | 4.0±2.2 | 4.3±4.1 | 4.1±3.6 | 0.962 |
| Previous DCC (%) | 13 (86.7) | 33 (60.0) | 17 (89.5) | 0.017 |
| **Echocardiography** |  |  |  |  |
| LAD (mm) | 54.9±4.3 | 53.9±2.6 | 58.1±4.8 | <0.001 |
| LA volume index (ml/m^2^) | 64.7±20.1 | 67.0±17.7 | 80.6±19.3 | 0.029 |
| LVEF (%) | 55.3±9.5 | 57.0±7.3 | 60.3±5.5 | 0.123 |
| **Medications at discharge (%)** |  |  |  |  |
| Class Ic drug | 5 (33.3) | 19 (14.5) | 2 (10.5) | 0.130 |
| Class III drug | 4 (26.7) | 27 (49.1) | 16 (84.2) | 0.003 |
| Beta-blocker | 4 (26.7) | 17 (30.9) | 7 (36.8) | 0.809 |
| Calcium channel blocker | 2 (13.3) | 3 (5.5) | 7 (36.8) | 0.003 |
| **Holter monitoring during follow up (%)** |  |  |  |  |
| 3-month visits | 10 (66.7) | 31 (56.4) | 11 (57.9) | 0.772 |
| 12-month visits | 8 (53.3) | 19 (34.5) | 6 (31.6) | 0.351 |
| At least once | 11 (73.3) | 37(67.3) | 11 (61.1) | 0.757 |
| **Recurrence (%)** |  |  |  |  |
| Early recurrence | 8 (53.3) | 29 (52.7) | 17 (89.5) | 0.015 |

AF, atrial fibrillation; BMI, body mass index; CBA, cryoballon ablation; DCC, direct current cardioversion; LA, left atrium; LVEF, left ventricular ejection fraction; PVAI, pulmonary vein antrum isolation; RFCA, radiofrequency ablation

**Supplemental Table 5**. **Baseline characteristics of subjects according to RFCA PVAI only, RFCA PVAI with additional ablation, CBA, and thoracoscopic maze group**

|  | **RFCA PVAI only**  **(n=17)** | **RFCA PVAI with additional ablation**  **(n=15)** | **CBA**  **(n=38)** | **Thoracoscopic maze (n=19)** | **P-value** |
| --- | --- | --- | --- | --- | --- |
| **Demographic data** |  |  |  |  |  |
| Age (years) | 65.8±5.5 | 62.3±5.2 | 64.8±6.0 | 59.8±8.9 | 0.021 |
| Men (%) | 8 (47.1) | 13 (86.7) | 34 (89.5) | 16 (84.2) | 0.003 |
| BMI (kg/m^2^) | 25.4±2.5 | 26.5±4.2 | 26.1±2.5 | 26.2±3.1 | 0.757 |
| **Past Medical History (%)** |  |  |  |  |  |
| Heart Failure | 2 (11.8) | 2 (13.3) | 8 (21.1) | 3 (15.8) | 0.816 |
| Hypertension | 15 (88.2) | 7 (46.7) | 28 (73.7) | 12 (63.2) | 0.066 |
| Diabetes mellitus | 6 (35.3) | 3 (20.0) | 10 (26.3) | 0 (0) | 0.053 |
| Thromboembolic events | 0 (0.0) | 1 (6.7) | 0 (0.0) | 3 (15.8) | 0.038 |
| Vascular events | 0 (0.0) | 0 (0.0) | 0 (0.0) | 0 (0) | N/A |
| CHA₂DS₂-VASc | 2.5±1.1 | 1.5±1.5 | 1.9±0.9 | 2.5±1.2 | 0.020 |
| **Atrial fibrillation history** |  |  |  |  |  |
| Years diagnosed with AF | 4.1±3.9 | 4.0±2.2 | 4.4±4.2 | 4.1±3.6 | 0.985 |
| Previous DCC (%) | 11 (64.7) | 13 (86.7) | 22 (57.9) | 17 (89.5) | 0.039 |
| **Echocardiography** |  |  |  |  |  |
| LAD (mm) | 54.2±3.1 | 54.9±4.3 | 53.8±2.4 | 58.1±4.8 | <0.001 |
| LA volume index (ml/m^2^) | 68.8±21.6 | 64.7±20.1 | 66.2±15.9 | 80.6±19.3 | 0.065 |
| LVEF (%) | 54.8±8.3 | 55.3±9.5 | 58.0±6.7 | 60.3±5.5 | 0.097 |
| **Medications at discharge (%)** |  |  |  |  |  |
| Class Ic drug | 8 (47.1) | 5 (33.3) | 11 (28.9) | 2 (10.5) | 0.114 |
| Class III drug | 6 (35.3) | 4 (26.7) | 21 (55.3) | 16 (84.2) | 0.003 |
| Beta-blocker | 7 (41.2) | 4 (26.7) | 10 (26.3) | 7 (36.8) | 0.654 |
| Calcium channel blocker | 1 (5.9) | 2 (13.3) | 2 (5.3) | 7 (36.8) | 0.008 |
| **Holter monitoring during follow up (%)** |  |  |  |  |  |
| 3-month visits | 8 (47.1) | 10 (66.7) | 23 (60.5) | 11 (57.9) | 0.707 |
| 12-month visits | 5 (29.4) | 8 (53.3) | 14 (36.8) | 6 (31.6) | 0.498 |
| At least once | 11 (64.7) | 11 (73.3) | 26 (68.4) | 11 (61.1) | 0.890 |
| **Recurrence (%)** |  |  |  |  |  |
| Early recurrence | 8 (47.1) | 8 (53.3) | 21(55.3) | 17 (89.5) | 0.033 |

AF, atrial fibrillation; BMI, body mass index; CBA, cryoballon ablation; DCC, direct current cardioversion; LA, left atrium; LVEF, left ventricular ejection fraction; PVAI, pulmonary vein antrum isolation; RFCA, radiofrequency ablation
